# Nonsteroid Anti‐Inflammatory Drug Use in Female Elite Ice Hockey Players With and Without Previous Concussions and Musculoskeletal Injuries

**DOI:** 10.1002/ejsc.12325

**Published:** 2025-05-31

**Authors:** Amanda Lahti, Anton Grundberg, Emelie Stenman, Kristina Sundquist

**Affiliations:** ^1^ Department of Clinical Sciences Malmö Center for Primary Health Care Research Lund University Malmö Sweden; ^2^ University Clinic Primary Care, Skåne University Hospital Region Skåne Sweden

**Keywords:** body checking, medications, NSAIDs, women’s ice hockey, women's sport

## Abstract

Ice hockey is a high‐intensity sport with high rates of concussions and musculoskeletal injuries. To manage pain, players may (over) use nonsteroid anti‐inflammatory drugs (NSAIDs) which can have adverse health effects. In 2022, the Swedish Women's Elite League (SDHL) became the first women's league to introduce body checking, potentially increasing injury risks and NSAID use. This study examines NSAID consumption among SDHL players and its associations with concussions and musculoskeletal injuries. All 224 players registered in SDHL 2023/2024 were invited to participate. The data of 158 included players (71%) aged (mean ± standard deviation) 23.4 ± 4.5 years were analyzed. NSAID use, concussions and musculoskeletal injury rates were assessed through a self‐reported questionnaire. In all players, 18% used NSAID at least once weekly, 15% used them one to three times per week, and 3% used them four to seven times per week. Players with previous concussions without persistent symptoms or musculoskeletal injuries did not have significantly higher odds of using more NSAIDs than those without. Players with persistent symptoms after concussion(s) were approximately five times more likely to use NSAIDs weekly than those with previous concussion(s) but without persistent symptoms (Odds Ratio (95% confidence interval): 4.9 (1.1, 21.3)) (*p* < 0.05). In conclusion, almost one‐fifth of female ice hockey players used NSAIDs weekly. Players with persistent post‐concussion symptoms are a subgroup that should be monitored more closely to prevent excessive NSAID use. The observed NSAID usage rates presented in this study represent important (baseline) reference values in female ice hockey players allowed to body check.

**Summary**

- 18% of female elite ice hockey players who are allowed to body check use NSAIDs regularly, from one to seven times per week, with no higher odds observed among those with concussions without persistent symptoms or musculoskeletal injuries.
- Players with persistent symptoms after concussion(s) have five times higher odds of using NSAIDs at least one time per week than those with previous concussion(s) without persistent symptoms. They may be a subgroup that should be monitored closely to prevent overuse of potentially harmful doses of NSAIDs.
- As women's ice hockey evolves with increased competition and body checking, NSAID use may change, warranting future studies to evaluate time trends in NSAID use, with this study providing important baseline reference values for such research.

## 1 Background

Non‐steroidal anti‐inflammatory drugs (NSAIDs) are commonly used for their anti‐inflammatory and pain‐relieving effects. As many NSAIDs are available over the counter and not classified as performance‐enhancing by the World Anti‐Doping Agency (WADA) (WADA 2024), athletes can easily access them. This ease of access, combined with athletes' willingness to compete despite pain and injuries (Mayer et al. 2018), has raised concerns about NSAID overuse and misuse in sports (Warden 2009; Davies 2021; Trinks et al. 2021; Tscholl et al. 2015; Gaetz 2022; Gorski et al. 2011).

Athletes frequently use NSAIDs to manage muscle soreness, facilitate recovery, and potentially enhance performance. However, current research does not support their efficacy for prophylactic use or performance enhancement (Roberts et al. 2024; de Oliveira et al. 2024). In male ice hockey, NSAIDs are the most commonly used medication, with usage rates double that of non‐players of the same age (Selanne et al. 2014). Elite male hockey players report higher medication use than those in other team sports (Gjelstad et al. 2023; Christensen et al. 2024), perhaps due to higher injury rates, which have also been observed in women's hockey. For example, the NCAA Injury Surveillance System (ISS) found that women's hockey had the highest concussion rates (0.91 per 1000 athlete‐exposures) among 16 collegiate sports, despite body checking not being allowed (Hootman et al. 2007).

Even if NSAIDs are not included in current guidelines for concussion management (Silverberg et al. 2020), it can be used in the acute inflammatory phase (Bergold 2016; Kalra et al. 2022). Although most concussion symptoms resolve, 10%–30% experience persistent or worsening symptoms, such as headaches, dizziness, and concentration issues (Renga 2021) that may continue for weeks, months, or even years. A higher number of concussions and being unconscious during the concussion increase the risk of persistent symptoms (Renga 2021). The condition lacks a specific treatment, so symptomatic management, sometimes including NSAIDs, is often used to alleviate symptoms (Renga 2021). Despite the widespread availability and frequent use of both prescribed and over‐the‐counter NSAIDs by athletes for various conditions, the use is not without risks.

NSAID use has been linked to serious health concerns in athletes, including kidney and liver damage (Chabbey and Martin 2019), exercise‐associated hyponatremia (Chabbey and Martin 2019), gastrointestinal ulcers (Papantoniou et al. 2023), gastric bleeding (Papantoniou et al. 2023) and cardiovascular problems (Tso et al. 2020). In addition, frequent users of NSAIDs have been found to have more positive attitudes toward general risk behaviors in terms of health and doping (Melzer et al. 2022). Additionally, NSAIDs may mask pain, allowing athletes to train and compete despite being hurt, potentially leading to delayed recovery and increased risk of injuries. In a high intensity contact sport like ice hockey, NSAID use may increase the risk of bleeding, potentially exacerbating complications in traumatic injuries, such as splenic ruptures and fractures, which are relatively common in the sport.

Females exhibit in general a greater response to painful stimuli and report higher levels of pain and chronic pain conditions than males (Christopher et al. 2020; Greenspan et al. 2007). Female athletes in handball, volleyball, and basketball have been shown to use more NSAIDs than their male counterparts (Christopher et al. 2020; Hager et al. 2021; John et al. 2023). However, NSAID use among female ice hockey players remains understudied. During the last decades, female ice hockey has grown rapidly in terms of the number of participants, spectators and financial resources—factors that all contribute to increased competition and evolution in sport (Norton and Olds 2001).

Additionally, the Swedish Elite Women's League, SDHL, became the first to allow body checking in 2022. Evidence from youth male ice hockey indicates that delaying the introduction of body checking led to a 50% reduction in overall injury rates and a 64% decrease in concussion rates (Black et al. 2017). It is thus reasonable to hypothesize that the introduction of body checking in women's ice hockey may similarly impact injury and concussion rates, potentially also influencing NSAID consumption among female ice hockey players.

This study aims to (1) evaluate the use of NSAIDs among elite female ice hockey players allowed body checking, (2) examine if players with previous concussions and musculoskeletal injuries have a higher use of NSAIDs than those without and, (3) evaluate if those with a higher number of concussions and/or those with persistent post‐concussion symptoms have a higher rate of NSAID use.

## 2 Method

This study uses baseline data from the *“Women's Ice Hockey Injury Study”*, a research project with the main purpose to examine injuries in the Swedish Elite Women's League, SDHL. This is the only women's league globally that allows body checking. The league has 10 teams, each with 20 to 25 players. SDHL representatives helped design the study and facilitated club contacts but did not access or analyze the data. In addition to injury data, the research project also collects comprehensive health data through an electronic questionnaire including medical history, prior injuries and medication use. Ice‐hockey variables such as player's position, team affiliation and ice‐hockey experience are also collected together with anthropometric and sociodemographic factors (including height, weight, BMI, income, and nationality).

An email invitation was sent to all 224 players registered in SDHL for the 2023/2024 season, with addresses obtained from the team sports managers. Only players aged 16 years and older were invited. Of the 224 players, 158 (71%) agreed to participate, including 21 players (13%) under 18 years old (range 16–37 years). A previous study's drop‐out analysis showed no age or height differences between participants and non‐participants (Lahti et al. 2024). Data collection occurred from December 2023 to April 2024, which is mid to late season. The players received research information and a survey link via email, detailing the study, procedures, risks, benefits, and ethical considerations. Participation was voluntary, with the option to withdraw at any time. All participants provided signed informed consent. The study adheres to the Declaration of Helsinki and was approved by the Swedish Ethical Review Authority. It is registered at ClinicalTrials.gov. Participants completed an online survey in Swedish or English, reporting age, height, and weight, with BMI calculated as weight (kg)/height (m)^2^. The survey included the following question on NSAID use:

1. How often do you use nonsteroidal anti‐inflammatory drugs (NSAID), including Ibuprofen, Ipren, Voltaren, Diklofenac etc? (never, less than one time per week, one to three times per week, four to seven times per week)

The survey included questions about current and previous concussions and musculoskeletal injuries (Appendix 1). Players were also classified based on whether they reported previous concussions (yes/no). Those with previous concussions were asked the following additional questions in the survey:

1. How many previous concussions have you had? (one, two, three or more)
2. Have you, on one or more occasions, been unconscious in connection with a concussion? (yes/no)
3. Do you have any lingering symptoms from your concussion(s)? (yes/no)
4. Please describe your lingering symptoms after your concussion(s):

Players reporting typical post‐concussion symptoms in question 4) such as headache, fatigue, vision changes, balance issues, confusion, dizziness, insomnia, irritability, apathy, personality changes, or concentration difficulties were classified as having persistent symptoms after a concussion (Permenter et al. 2024). Players with past injuries to the neck, back, upper, or lower extremities were classified as having a previous musculoskeletal injury; those without such injuries were classified as having none.

### 2.1 Statistical Analysis

We used SPSS (version 18.0, SPSS Inc., Chicago, IL, USA) for statistical analysis. Descriptive data are presented as means and standard deviations. Binary logistic regression was used to calculate odds ratios (OR) and 95% confidence intervals (95% CI) associated with NSAID use (ordinal dependent variable) and the presence of concussion, number of concussions, persistent symptoms after concussion(s), being unconscious during the concussion and musculoskeletal injury (binary predictor variable). The ordinal dependent variable was dichotomized into (1) *using NSAIDs less than one time per week* and (2) *using NSAIDs one to seven times per week*. We defined the second category as having a regular consumption of NSAIDs. All models were adjusted for age. The level of statistical significance for all tests was set at 0.05.

## 3 Results

**FIGURE 1:**
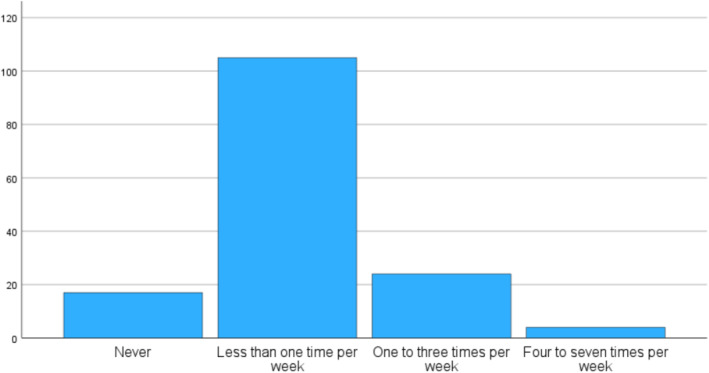
The weekly frequency of NSAID use among female Swedish elite ice hockey players allowed body checking.

Table 1 presents data on the 158 elite female ice hockey players, representing all 10 teams in the league and covering all player positions. Of the included players, 80 (51%) reported having a current or minimum one previous concussion, and 130 (82%) had experienced a musculoskeletal injury. Figure 1 illustrates the frequency of NSAID use among all players, whereas Table 2 presents the frequency of NSAID use stratified by players with a history of concussions and musculoskeletal injuries. Among all players, 18% used NSAIDs at least once weekly; 15% used them one to three times per week, 3% used them four to seven times per week. 77% of the players used NSAID less than once per week; 11% never used NSAIDs and 66% used NSAIDs once per week or less (5% missing). Table 3 shows the associations between NSAID use and previous injuries or concussions. There were no significant differences in NSAID consumption between players who had a minimum of one previous musculoskeletal injury versus no previous musculoskeletal injury, nor between those who had a minimum of one previous concussion versus no previous concussion. Neither did we find any significant differences in NSAID use between those who had two concussions or more versus only one concussion, or between those who had been unconscious during the concussion or not. However, players with persistent symptoms after concussion(s) were approximately five times more likely to use NSAIDs weekly than those with previous concussion(s) but without persistent concussion symptom(s) (OR (95% CI): 4.9 (1.1, 21.3)) (*p* < 0.05).

**TABLE 1:** Descriptive statistics of the included 158 female elite ice hockey players allowed body checking. Data is presented as mean ± SD or *n*, (%).

All players (*n* = 158)	
Age (years)	23.4 ± 4.5
Body height (cm)	169.1 ± 5.5
Body weight (kg)	68.4 ± 6.7
Body mass index (BMI)	23.9 ± 1.9
Have not had a previous concussion	70 (44)
Have had a previous concussion	80 (51)
Missing values	8 (5)
Number of concussions in those 80 players who have had a previous concussion	
One previous concussion	37 (46)
Two previous concussions	23 (29)
Three previous concussions	20 (25)
Unconsciousness in those 80 players who have had a previous concussion	
Not unconscious	65 (81)
Unconscious	15 (19)
Post‐concussion symptoms in those 80 players who have had a concussions	
No post‐concussion symptoms	71 (89)
Post‐concussion symptoms	9 (11)
Have not had a previous musculoskeletal injury	20 (13)
Have had a previous musculoskeletal injury	130 (82)
Missing values	8 (5)

*Note:* Data is presented as mean ± SD.

**TABLE 2:** NSAID use in elite female ice hockey players with and without previous concussion(s) and musculoskeletal injury.

NSAID use	All players (*n* = 158)	No previous concussion (*n* = 70)	Minimum one previous concussion (*n* = 80)	One previous concussion (*n* = 37)	Two previous concussions (*n* = 23)	Three previous concussions (*n* = 20)	Not unconscious during concussion(s) (*n* = 65)	Unconscious during concussion (*n* = 15)	No post‐concussion symptoms after concussion (*n* = 71)	Post‐concussion symptoms (*n* = 9)	No previous musculoskeletal injury (*n* = 20)	Minimum one previous musculoskeletal injury (*n* = 130)
Less than one time per week (*n*, %)	122 (77)	56 (80)	66 (83)	32 (87)	17 (74)	17 (85)	55 (85)	11 (73)	61 (86)	5 (56)	16 (80)	106 (82)
One to seven times per week (*n*, %)	28 (18)	14 (20)	14 (17)	5 (13)	6 (26)	3 (15)	10 (15)	4 (27)	10 (14)	4 (44)	4 (20)	24 (18)
Missing values (*n*, %)	8 (5)	0 (0)	0 (0)	0 (0)	0 (0)	0 (0)	0 (0)	0 (0)	0 (0)	0 (0)	0 (0)	0 (0)

**TABLE 3:** Odds ratios comparing NSAID use less than one time per week and using NSAIDs one to seven times per week in female elite ice hockey players with and without a previous musculoskeletal injury or concussion.

	OR (95% CI)	*p‐*value
Model 1		
Minimum of one previous musculoskeletal injury versus no previous musculoskeletal injury	0.9 (0.3, 3.0)	0.9
Model 2		
Minimum of one previous concussion versus no previous concussion	0.9 (0.4, 2.0)	0.7
Model 3		
Two concussions versus one concussion	0.9 (0.2, 5.1)	0.9
Three concussions versus one concussion	2.1 (0.4, 10.8)	0.4
Model 4		
Unconscious during concussion(s) versus not unconscious during concussion	2.1 (0.5, 8.0)	0.3
Model 5		
Having persistent symptom(s) after concussion(s) versus those with previous concussion(s) without having persistent symptom(s)	4.9 (1.1, 21.3)	0.04*

*Note:* All models are adjusted for age.

*p < 0.05.

## 4 Discussion

This study examined NSAID usage among elite female ice hockey players and associations with previous concussions and musculoskeletal injuries. We found that 18% of the players used NSAIDs regularly, from one to seven times per week. Notably, there was no significant association between increased NSAID use and the number of concussions or being unconscious during the concussion. Players with persistent symptoms after concussion(s) were approximately five times more likely to use NSAIDs weekly than those with previous concussion(s) but without persistent symptoms.

Studies suggest that in the general population, females exhibit a greater response to painful stimuli and report higher levels of pain and chronic pain conditions than males (Christopher et al. 2020; Greenspan et al. 2007). For example, one in five Germans aged 18–79 years use NSAIDs, paracetamol, or aspirin weekly, with women across all age groups consuming more painkillers than age‐matched men (Sarganas et al. 2015). In the general Spanish population aged 22–44 years, 18% use NSAIDs regularly (Gómez‐Acebo et al. 2018). In Sweden, 6% of the population aged 18 years or older used paracetamol and NSAIDs daily, and 16% once per week or several times during the week (Statista 2021).

The reported prevalence of NSAID use among athletes within different sports is widely ranging from 12% to 93%, with female athletes consuming more than their male counterparts (Trinks et al. 2021; Tscholl et al. 2015; Gorski et al. 2011; John et al. 2023; Harle et al. 2018; Warner et al. 2002). In male ice‐hockey players, data from doping controls reveal that 68% of all players took some kind of medication before the testing. 20% was NSAID and additional 22% other analgesics and antipyretics (Christensen et al. 2024). In a Finish study of young males, ice‐hockey players used more painkillers than age‐matched controls (18% vs. 10%), despite similarities in existing pain between the groups (Selanne et al. 2014). In the NHL, some players have reportedly been prescribed over 550 painkillers in one season, leading to overuse, medicalization, and problems with other substance abuse issues both during and after their careers.

Because of differences in measurement methods and study samples between various studies, we cannot draw any firm conclusions when comparing our results with male ice‐hockey players and the general population. However, the NSAID use among female ice‐hockey players that are allowed to body check seems to be at quite similar rates as in the general population and in male hockey players. Considering that females generally report higher pain levels (Christopher et al. 2020; Greenspan et al. 2007) and also may experience menstrual pain, higher NSAID use among female players than male players would be expected, but our results may not support this. These findings challenge assumptions about physical intensity, injuries, and NSAID use, contributing to discussions on whether body checking in other women's leagues and countries should be introduced.

Another finding in this study was that players with a previous concussion and persistent symptoms had five times higher odds of using NSAIDs regularly than those with previous concussion(s) but without persistent symptoms. This group may experience more pain, such as headaches and neck pain, which could lead to increased use of NSAIDs, either through self‐medication or prescription. Players who were unconscious during the concussion had 2.1 higher odds of using NSAIDs weekly than those who had a concussion but who were not unconscious. In addition, players with three concussions compared to one concussion also had 2.1 higher odds of using NSAIDs weekly. Even if none of these results were statistically significant, we cannot rule out a true effect due to the small sample size, reflected by the wide confidence interval. This question should therefore be evaluated in future studies.

Future studies should also examine the purpose of NSAID use among female ice hockey players, particularly those with post‐concussion symptoms. In football, precarious employment conditions such as having a short‐term contract increase pressure to play through injuries and associate to a higher analgesic use (Adams and Darby 2020; Roderick 2006; Roderick and Schumacker 2016). Similar sport‐related factors may influence usage in female hockey, but that remains to be examined. Future studies should also examine if there is a sport‐related prophylactic NSAID use, its role in injury treatment, and whether it is prescribed or self‐administered among female ice hockey players. As women's ice hockey continues to grow with increased participation, financial investments, and now introducing body checking in the sport and making it legal in Sweden, NSAID usage rates may change over time. We intend to follow this cohort longitudinally and examine these questions with this current study providing important baseline reference values for future research.

### 4.1 Strengths and Limitations

Study strengths include analyzing players from all SDHL teams and positions, reflecting a representative unique study population of the entire league. The dropout analysis showed no significant differences in body composition or age between participants and non‐participants, suggesting a representative cohort (Lahti et al. 2024). In this previous study (Lahti et al. 2024), there was no difference in body height, body weight or body mass index between players in SDHL and the North American Women's Elite League (PWHL), which makes it reasonable to assume that our results are applicable to other women's leagues that may consider allowing body checking in the future. A 71% response rate is also a strength. Even if this is the best‐recruited study population of female ice hockey players allowed body checking that is practically possible, it would still have been desirable to have larger study populations in the subgroup analyses, where we probably are unable to show statistically significant differences due to small sample sizes. Another limitation is that we did not examine when the injuries occurred or if it was sport‐related or associated with any pain condition. Despite this, the data is unique for this population of female elite ice hockey players allowed body checking, making the results noteworthy and can be a part of the decision making if bodychecking should be introduced for women in other countries. Females may use NSAIDs for menstrual symptoms, affecting sex comparisons, but this shouldn't be an issue when comparing female players with the general female population in similar ages. Further on, there are no gold standard for measuring NSAID consumption which makes it difficult to compare the usage with other study populations due to different methods. Another limitation is that data regarding previous concussions, musculoskeletal injuries and NSAID use is self‐reported and that players may underreport the use of NSAIDs due to the topic's sensitivity. Considering the use of self‐reported data regarding NSAID use, concussions, and musculoskeletal injuries, and the limited sample size, the results should be interpreted with caution. The study only covers NSAID use during the ice hockey season. If the usage is sport‐related, it may vary in on and off‐season. It would also be desirable to adjust the statistical analyses for the number of exposure hours, including both match and training, as players with more hours on the ice most likely also have a higher injury risk. Unfortunately, we did not have access to such data, which also is a limitation of this study.

## 5 Conclusions

In this study, 18% of female elite ice hockey players allowed body checking use NSAIDs regularly, from one to seven times per week, with no higher odds observed among those with concussions or musculoskeletal injuries. Players with a history of concussion(s) and persistent symptoms after the concussion have five times higher odds of using NSAIDs at least one time per week compared with those who had a previous concussion(s) but without persistent symptoms. This may be a subgroup that should be monitored more closely to prevent the overuse of potentially harmful use of NSAIDs. As women's ice hockey rapidly develops, NSAID use rates may change over time, warranting future studies to evaluate time trends and the purpose of NSAID use.

## Conflicts of Interest

The authors declare no conflicts of interest.

## Appendix 1

**Table:**

Do you have, or have you previously had any of the following conditions?		
Condition	Yes	No
Concussion		
Foot injury		
Lower leg injury		
Knee injury		
Thigh injury		
Hip injury		
Pelvis injury		
Back injury		
Shoulder injury		
Clavicula injury		
Neck injury		
Upper arm injury		
Elbow injury		
Forearm injury		
Hand injury		
Ligament injury		

## References

[ejsc12325-bib-0001] Adams, R. , and P. Darby . 2020. “Precarious Pursuits, Broken ‘Dreams’ and Immobility Among Northern Irish Soccer Migrants.” Sport in Society 23, no. 5: 920–937. 10.1080/17430437.2019.1593376.

[ejsc12325-bib-0002] Bergold, P. J. 2016. “Treatment of Traumatic Brain Injury With Anti‐Inflammatory Drugs.” Experimental Neurology 275, no. Pt 3: 367–380. 10.1016/j.expneurol.2015.05.024. 26112314 PMC6007860

[ejsc12325-bib-0003] Black, A. M. , B. E. Hagel , L. Palacios‐Derflingher , K. J. Schneider , and C. A. Emery . 2017. “The Risk of Injury Associated With Body Checking Among Pee Wee Ice Hockey Players: An Evaluation of Hockey Canada's National Body Checking Policy Change.” British Journal of Sports Medicine 51, no. 24: 1767–1772. 10.1136/bjsports-2016-097392. 28279963

[ejsc12325-bib-0004] Chabbey, E. , and P. Y. Martin . 2019. “Renal Risks of NSAIDs in Endurance Sports.” Revue Medicale Suisse 15, no. 639: 444–447. 30785678

[ejsc12325-bib-0005] Christensen, S. , A. Gjelstad , I. Björnsdottir , and F. Lauritzen . 2024. “Medicalization of Sport? A Mixed‐Method Study on the Use of Medications in Elite Ice Hockey.” Sports (Basel) 12, no. 1: 19. 10.3390/sports12010019. 38251293 PMC10818849

[ejsc12325-bib-0006] Christopher, S. , B. A. Tadlock , B. J. Veroneau , et al. 2020. “Epidemiological Profile of Pain and Non‐Steroid Anti‐Inflammatory Drug Use in Collegiate Athletes in the United States.” BMC Musculoskeletal Disorders 21, no. 1: 561. 10.1186/s12891-020-03581-y. 32814544 PMC7437034

[ejsc12325-bib-0007] Davies . November 10, 2021. “Addiction and Substance Abuse in the NHL—It’s Bigger Than the Game.” Hockey Writers.

[ejsc12325-bib-0008] de Oliveira, G. M. , F. A. Barcelos Andrade , A. B. Pereira , et al. 2024. “Is Physical Performance Affected by Non‐steroidal Anti‐inflammatory Drugs Use? A Systematic Review and Meta‐Analysis.” Physician and Sportsmedicine 52, no. 3: 207–216. 10.1080/00913847.2023.2220439. 37252825

[ejsc12325-bib-0009] Gaetz, M. 2022. “Substance Availability and Use in Ex‐Professional Ice Hockey Enforcers.” Scientific Reports 12, no. 1: 22204. 10.1038/s41598-022-26806-7. 36564454 PMC9789070

[ejsc12325-bib-0010] Gjelstad, A. , T. M. Herlofsen , A.‐L. Bjerke , F. Lauritzen , and I. Björnsdottir . 2023. “Use of Pharmaceuticals Amongst Athletes Tested by Anti‐doping Norway in a Five‐Year Period.” Frontiers in Sports and Active Living 5. 10.3389/fspor.2023.1260806. PMC10582642 37860156

[ejsc12325-bib-0011] Gómez‐Acebo, I. , T. Dierssen‐Sotos , M. de Pedro , et al. 2018. “Epidemiology of Non‐Steroidal Anti‐Inflammatory Drugs Consumption in Spain. The MCC‐Spain Study.” BMC Public Health 18, no. 1: 1134. 10.1186/s12889-018-6019-z. 30241493 PMC6150967

[ejsc12325-bib-0012] Gorski, T. , E. L. Cadore , S. S. Pinto , et al. 2011. “Use of NSAIDs in Triathletes: Prevalence, Level of Awareness and Reasons for Use.” British Journal of Sports Medicine 45, no. 2: 85–90. 10.1136/bjsm.2009.062166. 19666628

[ejsc12325-bib-0013] Greenspan, J. D. , R. M. Craft , L. LeResche , et al. 2007. “Studying Sex and Gender Differences in Pain and Analgesia: A Consensus Report.” Pain 132, no. Suppl 1: S26–s45. 10.1016/j.pain.2007.10.014. 17964077 PMC2823483

[ejsc12325-bib-0014] Hager, L. , B. Averbeck , C. Voelcker‐Rehage , and D. F. Kutz . 2021. “Sex Differences in the Consumption of Over‐The‐Counter Analgesics Among Amateur Volleyball Players.” BMC Sports Sci Med Rehabil 13, no. 1: 45. 10.1186/s13102-021-00273-5. 33910635 PMC8082781

[ejsc12325-bib-0015] Harle, C. A. , E. C. Danielson , W. Derman , et al. 2018. “Analgesic Management of Pain in Elite Athletes: A Systematic Review.” Clinical Journal of Sport Medicine 28, no. 5: 417–426. 10.1097/jsm.0000000000000604. 30156573

[ejsc12325-bib-0016] Hootman, J. M. , R. Dick , and J. Agel . 2007. “Epidemiology of Collegiate Injuries for 15 Sports: Summary and Recommendations for Injury Prevention Initiatives.” Journal of Athletic Training 42, no. 2: 311–319. 17710181 PMC1941297

[ejsc12325-bib-0017] John, J. M. , J. Bursik , C. Burgstahler , et al. 2023. “Prevalence of Sport‐Related Analgesic Use in German Elite Handball Players.” Deutsche Zeitschrift für Sportmedizin 74, no. 5: 168–174. 10.5960/dzsm.2023.568.

[ejsc12325-bib-0018] Kalra, S. , R. Malik , G. Singh , et al. 2022. “Pathogenesis and Management of Traumatic Brain Injury (TBI): Role of Neuroinflammation and Anti‐Inflammatory Drugs.” Inflammopharmacology 30, no. 4: 1153–1166. 10.1007/s10787-022-01017-8. 35802283 PMC9293826

[ejsc12325-bib-0019] Lahti, A. , A. Grundberg , E. Stenman , and K. Sundquist . 2024. “Physical Characteristics of Swedish Female Professional Ice Hockey Players Allowed Body Checking.” Journal of Strength and Conditioning Research. 10.1519/JSC.0000000000005009 PMC11841719 39630128

[ejsc12325-bib-0020] Mayer, J. , K. E. Giel , D. Malcolm , et al. 2018. “Compete or Rest? Willingness to Compete Hurt Among Adolescent Elite Athletes.” Psychology of Sport and Exercise 35: 143–150. 10.1016/j.psychsport.2017.12.004.

[ejsc12325-bib-0021] Melzer, M. , A.‐M. Elbe , and K. Strahler . 2022. “Athletes' Use of Analgesics Is Related to Doping Attitudes, Competitive Anxiety, and Situational Opportunity.” Frontiers in Sports and Active Living. 4. 10.3389/fspor.2022.849117. PMC9623021 36329852

[ejsc12325-bib-0022] Norton, K. , and T. Olds . 2001. “Morphological Evolution of Athletes over the 20th Century: Causes and Consequences.” Sports Medicine 31, no. 11: 763–783. 10.2165/00007256-200131110-00001. 11583103

[ejsc12325-bib-0023] Papantoniou, K. , C. Michailides , M. Bali , P. Papantoniou , and K. Thomopoulos . 2023. “Gastrointestinal Bleeding in Athletes.” Annals of Gastroenterology 36, no. 3: 267–274. 37144023 10.20524/aog.2023.0788 PMC10152804

[ejsc12325-bib-0024] Permenter, C. M. , R. J. Fernández‐de Thomas , and A. L. Sherman . 2024. Postconcussive Syndrome, StatPearls. StatPearls Publishing Copyright © 2024. StatPearls Publishing LLC. 30521207

[ejsc12325-bib-0025] Renga, V. 2021. “Clinical Evaluation and Treatment of Patients With Postconcussion Syndrome.” Neurol Res Int 2021: 5567695. 10.1155/2021/5567695. 34194843 PMC8181109

[ejsc12325-bib-0026] Roberts, B. M. , C. E. Sczuroski , A. R. Caldwell , et al. 2024. “NSAIDs Do Not Prevent Exercise‐Induced Performance Deficits or Alleviate Muscle Soreness: A Placebo‐Controlled Randomized, Double‐Blinded, Cross‐Over Study.” Journal of Science and Medicine in Sport 27, no. 5: 287–292. 10.1016/j.jsams.2024.02.002. 38383211

[ejsc12325-bib-0027] Roderick, M. 2006. “Adding Insult to Injury: Workplace Injury in English Professional Football.” Sociology of Health & Illness 28, no. 1: 76–97. 10.1111/j.1467-9566.2006.00483.x. 16509943

[ejsc12325-bib-0028] Roderick, M. , and J. Schumacker . 2016. “The Whole Week Comes Down to the Team Sheet’: A Footballer’s View of Insecure Work.” Work, Employment & Society 31, no. 1: 166–174. 10.1177/0950017016672792.

[ejsc12325-bib-0029] Sarganas, G. , A. K. Buttery , W. Zhuang , et al. 2015. “Prevalence, Trends, Patterns and Associations of Analgesic Use in Germany.” BMC Pharmacol Toxicol 16, no. 1: 28. 10.1186/s40360-015-0028-7. 26428626 PMC4591581

[ejsc12325-bib-0030] Selanne, H. , T. V. Ryba , K. Siekkinen , et al. 2014. “The Prevalence of Musculoskeletal Pain and Use of Painkillers Among Adolescent Male Ice Hockey Players in Finland.” Health Psychol Behav Med 2, no. 1: 448–454. 10.1080/21642850.2014.884463. 25750794 PMC4345898

[ejsc12325-bib-0031] Silverberg, N. D. , M. A. Iaccarino , W. J. Panenka , et al. 2020. “Management of Concussion and Mild Traumatic Brain Injury: A Synthesis of Practice Guidelines.” Archives of Physical Medicine and Rehabilitation 101, no. 2: 382–393. 10.1016/j.apmr.2019.10.179. 31654620

[ejsc12325-bib-0032] Statista . 2021. “How Often Do You Use Pain Killers Such as Alvedon, Ipren, Ibuprofen Etc?” [updated 2021‐01‐21; cited 2024 9th of September]. https://www.statista.com/statistics/732098/survey‐on‐usage‐of‐pain‐killers‐in‐sweden‐by‐frequency/.

[ejsc12325-bib-0033] Trinks, S. , A. B. Scheiff , M. Knipp , and A. Gotzmann . 2021. “Declaration of Analgesics on Doping Control Forms in German Football Leagues During Five Seasons.” Deutsche Zeitschrift für Sportmedizin 72, no. 2: 68–74. 10.5960/dzsm.2020.474.

[ejsc12325-bib-0034] Tscholl, P. M. , M. Vaso , A. Weber , and J. Dvorak . 2015. “High Prevalence of Medication Use in Professional Football Tournaments Including the World Cups Between 2002 and 2014: A Narrative Review With a Focus on NSAIDs.” British Journal of Sports Medicine 49, no. 9: 580–582. 10.1136/bjsports-2015-094784. 25878074 PMC4413681

[ejsc12325-bib-0035] Tso, J. , C. Hollowed , C. Liu , et al. 2020. “Nonsteroidal Anti‐Inflammatory Drugs and Cardiovascular Risk in American Football.” Medicine & Science in Sports & Exercise 52, no. 12: 2522–2528. 10.1249/mss.0000000000002404. 32520869 PMC7669570

[ejsc12325-bib-0036] WADA . 2024. World Anti‐doping Code International Standard Prohibited List 2024. edited by Montreal, Q. : Canada editor.

[ejsc12325-bib-0037] Warden, S. J. 2009. “Prophylactic Misuse and Recommended Use of Non‐Steroidal Anti‐Inflammatory Drugs by Athletes.” British Journal of Sports Medicine 43, no. 8: 548–549. 10.1136/bjsm.2008.056697. 19136504

[ejsc12325-bib-0038] Warner, D. C. , G. Schnepf , M. S. Barrett , D. Dian , and N. L. Swigonski . 2002. “Prevalence, Attitudes, and Behaviors Related to the Use of Nonsteroidal Anti‐Inflammatory Drugs (NSAIDs) in Student Athletes.” Journal of Adolescent Health 30, no. 3: 150–153. 10.1016/s1054-139x(01)00325-1. 11869920

